# Supplementary material for: Dynalign II: common secondary structure prediction for RNA homologs with domain insertions
Source: Nucleic Acids Res. 2014 Nov 21;42(22):13939–48. doi: 10.1093/nar/gku1172 (PMC4267632; doi:10.1093/nar/gku1172)
Supplement: SUPPLEMENTARY DATA [file supp_gku1172_nar-02021-z-2014-File012.zip › manual/Overview/Building.html]

RNAstructure Installation and Overview: Building the Repository


|  |  |  |
| --- | --- | --- |
|  | RNAstructure Installation and Overview Building Instructions | - Contents - Index |
| General Build Information  Do I need to build the package?  RNAstructure is provided both as source code and as prebuilt executables. Prebuilt executables are currently available for 32-bit Linux, 64-bit Linux, Max OS-X (Leopard and higher), 32-bit Windows, and 64-bit Windows. If you are using one of these operating systems, then you probably do not need to build the package. The available executables will work fine.   ---  Building the GUI Interfaces  Building the RNAstructure JAVA GUI  In the RNAstructure directory, open the file "compiler.h". This file contains compiler flags and Java include paths that must be activated to match the current operating system. See the Building Requirements page for details.  Then, in the RNAstructure/RNAstructure\_java\_interface directory, activate the Makefile by typing "make." When the JAVA GUI is built correctly, two new files will exist in the RNAstructure/exe directory: a single large dynamic RNAstructure library (whose name depends on the operating system), and a JAVA JAR archive, called RNAstructure.jar. See Running the RNAstructure JAVA GUI for details on how to use these files.  Building the RNAstructure Windows native GUI  RNAstructure requires Microsoft Visual Studio 2010 AND the Intel C++ compiler for compilation.  Use the solution file, RNAstructure.sln, in RNAstructure/RNAstructure\_windows\_interface.   ---  Building the Command Line (Text) Interfaces  Configuring the Build on Unix/Linux/OS-X  The command line (text) interfaces for RNAstructure are built using the Makefile in the main directory (RNAstructure/). The definitions for compiler and flags are in RNAstructure/compiler.h. This file is provided with the defaults set for compiling on Linux using g++. To change the platform and/or compilers, this file should be manually edited. Example flags are provided for Linux, Mac, and Cygwin platforms. To change the operating system to a non-Linux system, simply remove the "#" symbol (which denotes comments) from the variable definitions under the target type. Also, add "#" in front of the Linux flags.  Note also that flags for building with OpenMP can be set (CXXOPENMPFLAGS). The default flags will work for Linux and Mac using g++ and g++/LLVM, respectively. An example flag set is also provided for Intel compilers, and this will need to used if Intel compilers are being used.  Building on Unix/Linux/OS-X  To build the package, use the command "make all" in RNAstructure/. The command line executables are built and placed in RNAstructure/exe/. You should probably then update your PATH variable to include this directory.  Building CUDA on Unix/Linux/OS-X  In adition, if you have a CUDA compiler, you can build partition-cuda by typing "make cuda" in the RNAstructure directory. This can then be run if you have a CUDA-capable graphics card.  An optional "make install" command will copy all the executables to the /usr/local/RNAstructure/ directory. This is helpful in a shared computing environment. This command can only be executed as root.  Building Serial and SMP on Unix/Linux/OS-X  Dynalign, Fold, Multilign, and TurboFold can be built in SMP form, which allows them to delegate their workload across multiple processors at once in a shared memory environment.  By default, "make all" will build the serial programs and their SMP counteparts. To build only the serial programs, for example if the compiler does not have OpenMP support, "make serial" can be used with the Makefile in the main directory (RNAstructure/). To build only the SMP programs, use the "make SMP" target.  Building Multifind  Multifind is built separately from the rest of the package. After setting the INCLUDESVM variable, it can be built with the command make Multifind or make Multifind-smp.  Building on DOS/Windows  For DOS/Windows command line (text) interfaces, Microsoft Visual Studio (2010) AND the Intel C++ compiler are required. Use Visual Studio to open the solution called RNAstructure\RNAstructure\_windows\_text\_interfaces\RNAstructure\_windows\_text\_interfaces.sln. Then build using Visual Studio. The executables will be depositing in the RNAstructure\RNAstructure\_windows\_text\_interfaces\Release folder.   --- | | |
| Visit The Mathews Lab RNAstructure Page for updates and latest information. | | |
